# Population structure and inter-species admixture within a likely extinct yet formerly widespread Hawaiian honeycreeper

**DOI:** 10.1098/rsbl.2025.0265

**Published:** 2025-10-29

**Authors:** Natalia A. S. Przelomska, Michael G. Campana, Helen F. James, Logan Kistler, Nancy Rotzel McInerney, Oscar A. Pérez-Escobar, Molly Hagemann, Jim J. Groombridge, Robert C. Fleischer

**Affiliations:** ^1^ Center for Conservation Genomics, Smithsonian's National Zoo and Conservation Biology Institute, Smithsonian Institution, Washington, DC, USA; ^2^ Department of Anthropology, National Museum of Natural History, Smithsonian Institution, Washington, DC, USA; ^3^ School of the Environment and Life Sciences, University of Portsmouth, Portsmouth, UK; ^4^ Department of Vertebrate Zoology, National Museum of Natural History, Smithsonian Institution, Washington, DC, USA; ^5^ Royal Botanic Gardens, Kew, Richmond, UK; ^6^ Vertebrate Zoology Department, Bernice Pauahi Bishop Museum, Honolulu, HI, USA; ^7^ Durrell Institute of Conservation and Ecology, School of Natural Sciences, University of Kent, Canterbury, UK

**Keywords:** admixture, Hawaiian honeycreepers, island biogeography, morphology, museum specimens, population genomics

## Abstract

The Hawaiian honeycreepers simultaneously represent one of the most spectacular avian adaptive radiations and are one of the most endangered avian groups. This clade’s few geographically widespread species can serve as a model to understand population-level processes shaping differentiation and characterizing decline. One such species is the likely extinct ʻōʻū (*Psittirostra psittacea*), a parrot-like beaked honeycreeper with a frugivorous feeding ecology. We compiled morphological and hybridization-captured ancient DNA datasets for the ʻōʻū from museum specimens from across the Hawaiian archipelago. We find (i) genomic differentiation among ʻōʻū from Kauaʻi, Lānaʻi, and the remaining Hawaiian Islands and (ii) a larger phenotype on Kauaʻi and smaller Maui Nui morphological phenotypes. While the differentiated population on Kauaʻi is likely a result of Kauaʻi’s geographical isolation, the divergent population on Lānaʻi is harder to explain by biogeography alone. Thus, we investigated whether the unexpected divergence of Lānaʻi ʻōʻū could be attributed to inter-species admixture with the geographically overlapping, now extinct ‘parrot-billed’ Lānaʻi hookbill (*Dysmorodrepanis munroi*) or a critically endangered Maui endemic, the kiwikiu (*Pseudonestor xanthophrys*). We detect significant admixture between the Lānaʻi ʻōʻū population and the Lānaʻi hookbill, possibly explaining the observed population structure and associating interspecific breeding with populations on the precipice of extinction.

## 1. Introduction

The Hawaiian Islands are the world’s most isolated oceanic archipelago and, as such, are a prominent model system for studies in ecology and evolution. This ‘living laboratory’ has greatly enhanced our understanding of adaptive radiations [1–4] and extinction, phenomena exemplified by the Hawaiian honeycreepers (Fringillidae: Drepanidinae) [5–7]. Hawaiian honeycreepers exhibit high variation in osteological morphology, feeding niche, plumage and song [8,9]. Unfortunately, about two thirds of known Hawaiian honeycreeper species have gone extinct since humans’ arrival to the archipelago around AD 1000−1200 [10], including at least 22 species extinct since European arrival in Hawaii and another 18 only known from subfossils [11–14]. The International Union for Conservation of Nature classifies 14 of 17 extant drepanidine species as critically endangered, endangered or vulnerable [15].

Most drepanidine lineages exhibit morphological differentiation and little or no gene flow across the Hawaiian Islands, resulting in the designation of island-specific species and subspecies [5,11]. However, at least three species occurred on all major islands (Kauaʻi, Oʻahu, Molokaʻi, Maui, Lānaʻi and Hawaiʻi), with no currently recognized taxonomic splitting: iʻiwi (*Vestiaria coccinea*), ʻapapane (*Himatione sanguinea*) and ʻōʻū (*Psittirostra psittacea*) [5,16]. The iʻiwi and the ʻapapane remain extant on all major islands (the exception being the extirpation of iʻiwi from Lānaʻi), while the ʻōʻū is probably extinct [17,18]. Feeding ecology is an important driver in the radiation and can be associated with dispersal at the species level and intraspecific connectivity of populations. The iʻiwi and the ʻapapane are primarily nectivorous, whereas the ʻōʻū has a diverse diet but is primarily frugivorous. Thus far, rigorous analyses addressing intraspecific morphological and genetic differentiation have not yet been conducted for any of these three honeycreepers, limiting our understanding of how population-level processes shape the diversity and characterize the decline of widespread species in this radiation.

The ʻōʻū was a heavy-bodied Hawaiian honeycreeper with olive-green plumage and a dappled green (female) or yellow (male) head. It had a thick, parrot-like beak [9], a phenotype shared with only two other drepanidine species: the kiwikiu (*Pseudonestor xanthoprys*) of Maui and Molokaʻi and the Lānaʻi hookbill (*Dysmorodrepanis munroi*), known from Lānaʻi only. Before its disappearance, the ʻōʻū’s behaviour was recorded in both traditional ecological knowledge [19] and in observations collected by naturalists and ornithological collectors. The ʻōʻū was observed in mid-elevation mesic to wet ʻōhiʻa forests, primarily feeding on ʻieʻie (*Freycinetia arborea*; Pandanaceae) vine fruits, ʻōhiʻa (*Metrosideros polymorpha*; Myrtaceae) nectar and other native fruits such as ʻōhā (*Clermontia* spp.: Campanulaceae) [8,20]. A strong flier, it was observed to travel in small groups between feeding grounds, depending on the seasonal availability of fruits [9]. Naturalists collected a number of these birds, and some noted minor differences in plumage or size across the archipelago [21–23], differences that are not currently regarded as sufficient for designating subspecies.

Naturalists frequently recorded the ʻōʻū in the 1800s (e.g. [9]). A 1976−1981 population survey estimated a census size of 400 ± 300 individuals on Hawaiʻi and approximately nine individuals on Kauaʻi [24]. The ʻōʻū’s last confirmed sighting on Hawaiʻi was in 1987 after a lava flow from Mauna Loa destroyed much of the available ʻōʻū habitat [17]. The final ʻōʻū stronghold was the Alakaʻi swamp on Kauaʻi, where the last accepted sighting was in February 1989 [25]. Sound recordings suggest the Kauaʻi population may have persisted for a couple more years, but no more records were reported following Hurricane Iniki in 1992.

Here, we use morphological and DNA sequence data derived from museum specimens to investigate population structure, diversity and inbreeding within the likely extinct, formerly widespread ʻōʻū. Furthermore, given our unexpected discovery of genomically distinct ʻōʻū individuals from Lānaʻi, we investigated the potential for inter-species admixture across the ‘parrot-billed’ honeycreepers.

## 2. Materials and methods

### (a) Morphological dataset and analysis

We measured wing chord, culmen length, culmen width, tarsus length and tail length from 89 museum collection study skins in adult plumage (electronic supplementary material, table S1). We excluded culmen width due to a high proportion of missing data (26%). We used ANOVA to assess sexual dimorphism and inter-island morphological variation. To explore variation that could have been obscured by differing island sample sizes, we also applied a permutation approach, running ANOVA on down-sampled island groups. We then applied linear discriminant analysis (LDA) in the R [26] package MASS 7.3-60 [27] to examine whether detected differences in the phenotypic data could be specifically attributed to sex and inter-island differentiation, respectively (for further details, see electronic supplementary material).

### (b) DNA sampling and extraction

A total of 50 toe pad, skin or bone samples representing 49 *Psittirostra psittacea* individuals were obtained from specimens in collections of the American Museum of Natural History (AMNH), the British Museum (BM), the Bernice Pauahi Bishop Museum (BPBM), the Academy of Natural Sciences Drexel University, Philadelphia (ANSP), Muséum National d’Histoire Naturelle, Paris (MNHN), Statens Naturhistoriske Museum, Copenhagen (ZMK), Royal Ontario Museum (ROM), the Museum of Vertebrate Zoology at Berkeley (MVZ) and the Museum of Zoology, University of Cambridge (UMZC) (electronic supplementary material, table S2). AMNH 176717 was sampled twice. Samples were chosen to reflect as broad a distribution across the Hawaiian Islands as possible. For most samples, we used a sterile scalpel to shave an approximately 2 × 2 mm piece of toe pad from each specimen. For a subset of samples, a small piece of dried skin from the abdominal incision site or a small piece of bone was removed from the specimen.

Eighteen samples were extracted in an ancient DNA (aDNA) laboratory at the University of Durham. The remaining samples were extracted in the aDNA laboratory of the Centre for Conservation Genomics, Smithsonian’s National Zoo and Conservation Biology Institute (CCG-NZCBI), Washington, DC, USA. We practised stringent aDNA protocols to limit sample contamination and extracted samples according to a previously published protocol [28] (electronic supplementary material).

Given unexpected differentiation of three ʻōʻū individuals from Lānaʻi (see Results), we investigated the possibility of admixture with two geographically overlapping, ‘parrot-billed’ Hawaiian honeycreeper species: the kiwikiu and the Lānaʻi hookbill. Hybridization between these species has been suggested previously based on the Lānaʻi hookbill’s morphology and rarity [29]. The kiwikiu sample was derived from a modern tissue sample salvaged from the kiwikiu captive breeding programme (BPBM 185328). We obtained a toepad sample from the singular Lānaʻi hookbill specimen (BPBM 4792), an individual collected in 1913. The Lānaʻi hookbill sample was extracted in the CCG-NZCBI aDNA laboratory as described above.

### (c) DNA library preparation and high-throughput sequencing

The initial 18 samples were prepared using KAPA LTP library preparation kits and indexed with iNext primers [30], followed by hybridization-captured using a custom 40 000 drepanidine single nucleotide polymorphism (SNP) bait set (myBaits^®^: Daicel Arbour Biosciences, Ann Arbour, MI, USA) [31,32]. After evaluation of results, a more efficient library preparation strategy was adopted—the blunt end single tube (BEST) method [33] for the remaining samples. Nine samples were prepared using both KAPA and BEST. The kiwikiu sample was built into KAPA libraries as above. We built a single-stranded library from the Lānaʻi hookbill DNA using the SRSLY^®^ Pico kit (Claret Bioscience, Scotts Valley, CA, USA) (for further details, see electronic supplementary material).

### (d) DNA read processing and single nucleotide polymorphism calling

Adapter sequences and low-quality bases were trimmed from each ʻōʻū sample’s reads, and overlapping reads were merged using AdapterRemoval 2.1.7 [34] with the following parameters: --trimqualities --minquality 20 --minalignmentlength 25 --minlength 25 --collapse. Trimmed and merged sequences were then aligned to the draft reference sequence available for another Hawaiian honeycreeper species—the Hawaiʻi ʻamakihi (*Chlorodrepanis virens*; GenBank: GCA_003286495.1 [31]) using BWA 0.7.17 *aln* [35] with the seed disabled [36]. Polymerase chain reaction duplicates were removed using SAMtools 1.9 [37,38]. We estimated cytosine deamination rates and authenticated aDNA damage profiles using MapDamage 2.0.8 [39]. Based on the damage profiles, we masked the terminal two nucleotides of the BEST libraries and the terminal three nucleotides of the KAPA libraries’ sequences. For individuals with multiple libraries, we merged the masked alignments using SAMtools. We calculated the number of unique mapped reads and the number of on-target unique mapped reads using SAMtools 1.18 and BEDtools 2.31.0 [40]. We retained 28 ʻōʻū individuals for which we recovered at least 100 000 on-target unique reads.

For the inter-species admixture investigation, we used the ‘akikiki (*Oreomystis bairdi*; SRA: SRR8746532 [41]) as a closely related outgroup to the ‘parrot-billed’ honeycreepers [42]. The kiwikiu, ‘akikiki, and Lāna‘i hookbill samples were processed following the same pipeline as the ʻōʻū samples except that they were processed with more recent versions of the pipeline software (AdapterRemoval 2.3.2, SAMtools 1.13 and MapDamage 2.2.1) and the modern kiwikiu and ‘akikiki samples were not masked for DNA damage. Based on the Lānaʻi hookbill’s MapDamage profile, we masked its sequences’ three terminal nucleotides.

For the 28 retained ʻōʻū individuals, we excluded sequences with mapping qualities <25 and called variants using BCFtools 1.18 [38] *mpileup* and *call* (multi-allelic caller model). Using VCFtools 0.1.16 [43], we included only localized, biallelic, autosomal SNPs with no more than 5% missing data per site and minimum minor allele frequencies of 5%. To explore the impact of missing data and allelic drop-out on our population genomic inferences, we filtered data for minimum depths of 1×, 3×, 5× and 10× per site per individual. For the analyses of possible inter-species admixture, we repeated the variant-discovery and filtration pipeline adding the kiwikiu, ‘akikiki and Lānaʻi hookbill samples.

### (e) Population structure, genetic diversity and inbreeding

For each ʻōʻū dataset, we assessed population structure through principal component analysis (PCA) using PCAngsd [44] and *K*-means clustering using ADMIXTURE [45] and NGSadmix [46] (electronic supplementary material). We repeated these analyses accounting for linkage disequilibrium (LD) and putative kin (electronic supplementary material). Further, we repeated a subset of the PCAs using BEAGLE-format likelihoods and did not observe any differences to the results, supporting that these are robust at the genotype depths analysed. Based on the above analyses, we assigned likely populations of origin for two unprovenanced individuals (ANSP 3357 and ANSP 3359). To explore potential inter-island differences and population genomic health, we computed observed and expected homozygosities and per-individual inbreeding coefficients for the 5× and 10× minimum depth datasets using PLINK 2.00a6 [47].

### (f) Inter-species admixture

Based on the population structure results, we defined three ʻōʻū populations: a Kauaʻi population, a Lānaʻi population and a population consisting of individuals from the islands of Hawaiʻi, Maui, Molokaʻi and Oʻahu (hereafter ‘HMMO’ population). For the inter-species admixture analyses, ANSP 3357 and ANSP 3359 were assigned to the Kauaʻi and HMMO populations, respectively. We calculated *D-* and *f*-branch statistics using Dsuite [48] and explored admixture graphs automatically using AdmixTools 2.0.7 [49], allowing up to two admixture edges. Since some ADMIXTURE and NGSadmix analyses clustered individuals from Hawaiʻi, we repeated the Dsuite and AdmixTools analyses, separating individuals from Hawaiʻi from the remaining HMMO individuals.

## 3. Results

### (a) Morphological differentiation

**Figure 1.**
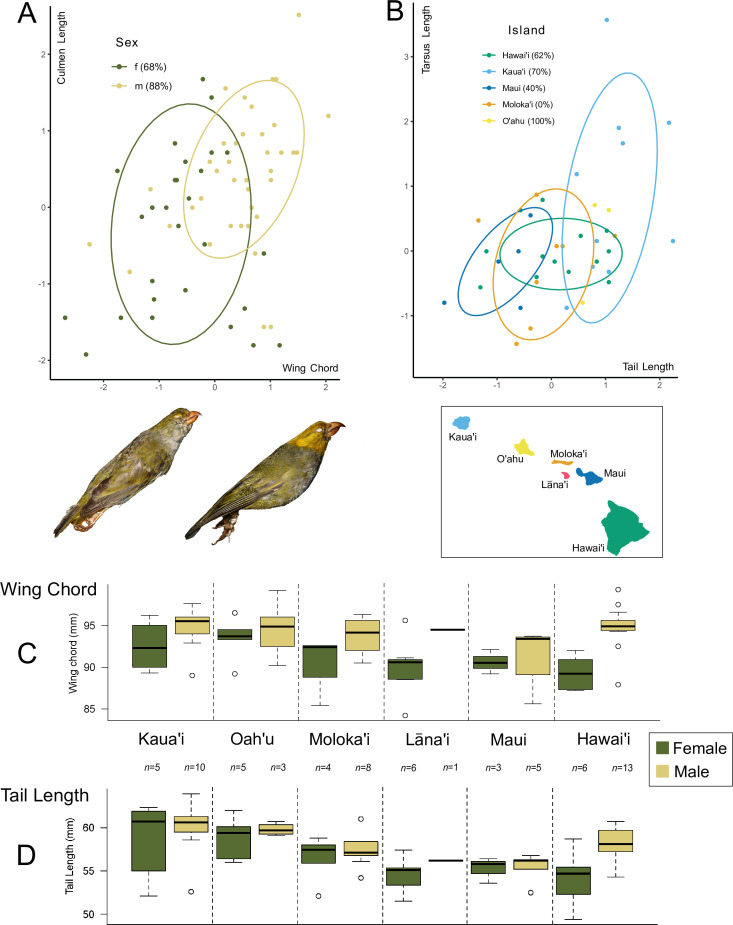
Morphometrics based on ʻōʻū specimen measurements split by sex and island group. (A) Culmen length against wing chord differentiating sexes. Ellipses represent 75% confidence intervals. Percentages in the legend represent posterior probabilities of assignment to this group based on LDA on all four analysed traits. (B) Tarsus length against tail length differentiating among islands (male ʻōʻū only). Ellipses represent 68% confidence intervals. Percentages in the legend represent posterior probabilities of assignment to this group based on LDA on all four analysed traits. (C) Boxplots summarizing the wing chord dataset. (D) Boxplots summarizing the tail length dataset. Inset photographs in A (© University Museum of Zoology, University of Cambridge): female ʻōʻū (left) and male ʻōʻū (right).

The ANOVA on the data split by sex revealed significantly smaller measurements for female birds compared to male birds for the following traits: wing chord, culmen length and tail length (*p* < 0.003), but not for tarsus length (figure 1A,C,D, electronic supplementary material, table S3 and figure S3). The LDA for classification to the correct sex based on phenotypic data resulted in successful predictions for 68% of female birds and 88% of male birds, where wing chord had the highest discriminatory value, followed by culmen length. The dimorphism justified our decision to separate sexes in the tests for inter-island differentiation. Significant inter-island differentiation emerged from the ANOVA tests for tail length and wing chord among male birds (*p* < 0.019) and no traits among the female birds. For wing chord, Maui birds are smaller than those from Hawaiʻi and Kauaʻi and for tail length, Maui, Molokaʻi and Hawaiʻi specimens are smaller than those from Kauaʻi and Maui specimens are smaller than those from Oʻahu (Tukey’s honestly significant difference (HSD) test adjusted *p* < 0.013 for all significant instances) (figure 1B,C,D, electronic supplementary material). Although not a replacement for larger sampling sizes, our down-sampled permutations of the data served to demonstrate robustness of the above and indicated smaller culmen length in both females (20% iterations where *n* = 3 for island groups) and males (30% iterations where *n* = 3 for island groups) on Oʻahu. For further details, see electronic supplementary material results, table S4, and figure S3. The LDA for classification to island group based on phenotype for males resulted in two discriminant functions, accounting for 72.2% and 17.5% of the variation, respectively. Tail length had the highest discriminatory value, followed by tarsus length.

### (b) DNA extraction and sequencing

For the 49 sequenced ʻōʻū individuals, we obtained between 2081 and 5 945 326 unique mapped endogenous reads (mean ± s.d.: 1 322 208 ± 1 460 084), of which 270 to 972 936 fell within the bait-targeted regions (mean ± s.d.: 277 931 ± 323 021) (electronic supplementary material, table S5). For the kiwikiu, Lānaʻi hookbill and ‘akikiki samples, we obtained 1 571 614, 6 136 567, and 5 366 968 unique mapped reads, respectively. Of these, 210 252 kiwikiu, 1 546 263 Lānaʻi hookbill, and 1 105 830 ‘akikiki reads mapped to the targeted regions.

We retained 44 273, 9596, 3606 and 737 biallelic SNPs after quality filtering in the 1×, 3×, 5× and 10× minimum depth ʻōʻū datasets, respectively. LD pruning reduced these datasets to 20 221, 4487, 1778 and 430 sites, respectively. For the inter-species admixture analyses, we retained 27 443, 6633, 2673 and 546 SNPs (13 164, 3231, 1350 and 334 after LD pruning) for the 1×, 3×, 5× and 10×minimum depth datasets, respectively. After filtering for depth, residual per-individual missingness was low across all datasets (maximum 18.1%; electronic supplementary material, tables S6–S9).

### (c) Population genomic structure

The first two to three axes of the PCA (figure 2A, electronic supplementary material, figures S6–S29) indicate clear population structure. PC1 separates Kauaʻi individuals from the remaining ‘ōʻū. PC2 and/or PC3 separates out some Lānaʻi individuals. Samples from the other islands form a more compact cluster (HMMO population). Within the HMMO population, we observe possible substructure between the Oʻahu, Maui, Molokaʻi and Hawaiʻi individuals. The unprovenanced ‘ōʻū individuals cluster with the Kauaʻi (ANSP 3357) and HMMO (ANSP 3359) individuals. The two-population ADMIXTURE and NGSadmix models separate Kauaʻi from all other samples, while the three-population and higher models also frequently distinguish the Lānaʻi and Hawai‘i populations (electronic supplementary material figures S30-77).

**Figure 2.**
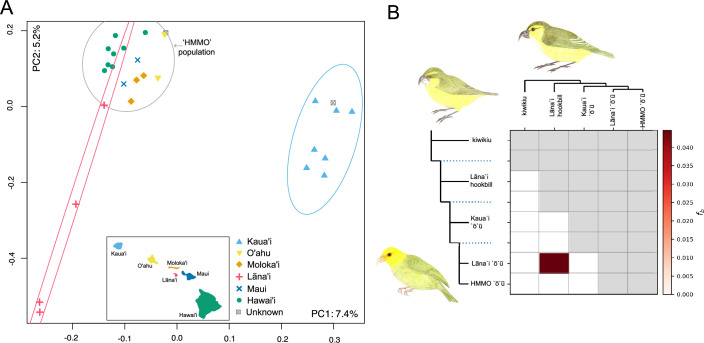
(A) Principal component analysis of the 3× minimum depth ʻōʻū dataset showing separation of Kaua'i from other island samples on PC1 and separation of most Lāna'i samples on PC2. Ninety-five per cent data ellipses are drawn for Kaua'i (light blue), Lāna'i (red) and other islands (grey), with a map of the Hawaiian Islands. (B) *f*-branch co-ancestry between Lāna'i ʻōʻū and Lāna'i hookbill in the unpruned 3× minimum depth dataset. Inset illustrations (clockwise from bottom left): male ʻōʻū, kiwikiu, Lāna'i hookbill (Angela Przelomski).

### (d) Genetic diversity and inbreeding

All ʻōʻū individuals were outbred, with no obvious patterning of homozygosity or inbreeding values across islands (electronic supplementary material, tables S10–S11).

### (e) Inter-species admixture

Dsuite *D*, *f_4_-*ratio and *f*-branch statistics found strong evidence of co-ancestry between the Lāna‘i hookbill and Lāna‘i ʻōʻū population (figure 2B, electronic supplementary material, figures S78–S84, table S12). AdmixTools modelling agreed with the Dsuite results (electronic supplementary material, figures S85-108). Models without admixture edges had poor fits (strongly rejecting the null), while allowing admixture significantly improved model fit (electronic supplementary material, table S13). In 29 of 32 (91%) models including one or two admixture edges, the Lāna‘i hookbill was modelled as a mixture of Lāna‘i ʻōʻū-like ancestry and an ancestry that was basal to ‘ō‘ū (electronic supplementary material). The inferred second admixture edge was not replicable between datasets.

## 4. Discussion

The likely extinct ʻōʻū was known for its migratory behaviour [9], driven by the phenology of fruiting plants, which suggests sustained intraspecific gene flow in spite of its widespread distribution across the Hawaiian Islands. Nonetheless, our population-level analysis of the ʻōʻū revealed geographical structure with a genomically and morphologically divergent population on Kauaʻi as well as a genomically distinct population on Lānaʻi and morphologically smaller individuals from Maui Nui. Kauaʻi is the oldest of the main Hawaiian Islands (emerging approx. 5 Ma and existing in isolation for at least 1 Ma) and the most geographically separated [50], which may be the primary cause of the genomic and morphological differentiation [51]. For instance, Campillo [52] also noted greater divergence of Kauaʻi versus other populations of ‘apapane. The divergence of the Kauaʻi ʻōʻū population is consistent with expectations from biogeography and may reflect incipient reproductive isolation and the initiation of speciation within *Psittirostra* as has been observed in other drepanidine genera [32].

The Lānaʻi differentiation pattern was unexpected as this island is geographically close to Maui, a larger island for which the ʻōʻū fell within the HMMO cluster. Furthermore, the Maui Nui island group of Maui, Lānaʻi, Molokaʻi and Kaho‘olawe comprised a single island until approximately 200 ka with intermittent land connections between these islands (except Kaho‘olawe) existing until the end of the last glacial maximum (approximately 20 ka) [53]. This suggests a Holocene origin of the Lānaʻi population divergence, not supporting the pattern of differentiation observed. Our results document admixture between the Lānaʻi ʻōʻū population and the Lānaʻi hookbill, which could explain this unexpected population structure.

Admixture appears to be rare in Hawaiian honeycreepers [54]. However, admixture in closely related avian species has been documented during population declines (e.g. [55]). The Lānaʻi hookbill was only sighted four times between 1913 and 1918 and collected only once, indicating its extreme rarity by the early twentieth century [56]. Given its decline to extinction at the time, hybridization between the hookbill and the closely related Lānaʻi ʻōʻū is likely. AdmixTools modelled gene flow from the Lānaʻi ʻōʻū into the Lānaʻi hookbill. However, the opposite geneflow direction (from Lānaʻi hookbill into Lānaʻi ʻōʻū) is required if the Lānaʻi ʻōʻū’s population divergence resulted from Lānaʻi hookbill introgression. Nevertheless, we cannot rule out bidirectional introgression from our data. Further, we cannot currently identify the source of non-‘ō‘ū gene flow into the Lāna‘i hookbill, as we did not exhaustively analyse potential admixing species here. Taxonomically comprehensive, genome-wide analyses will be necessary to establish whether this introgression derives from a ghost lineage. Our findings of admixture into the Lāna‘i hookbill also revive the debate around its taxonomic status (unique species or hybrid) [23], which similarly could be resolved by more in-depth genomic analyses.

Our study documents gene flow between two species of Hawaiian honeycreepers during and prior to their Late Holocene declines, suggesting that interspecific admixture can be promoted in regions undergoing an extinction event. Furthermore, our population-level analysis of the ʻōʻū adds to the evidence that Hawaiian honeycreeper species tend to harbour remarkably high levels of genetic diversity, regardless of inbreeding status, which is generally reflective of their historically large population sizes [57]. The demise of this once widespread species signifies the loss of Hawaiian honeycreeper evolutionary potential and, more specifically, of an element of functional diversity: the ʻōʻū was more strongly frugivorous than any other Hawaiian honeycreeper species whose diet is known.

## Data Availability

Raw genetic sequence data have been deposited in NCBI (Bioproject PRJNA1244941). Raw morphological measurement data is available in electronic supplementary material, table 1 (in ESM file 2). Supplementary material is available online [58].
